# Histiocytic Sarcoma: Challenging Course, Dismal Outcome

**DOI:** 10.3390/diagnostics11020310

**Published:** 2021-02-15

**Authors:** Kim Francis Andersen, Lene Dissing Sjö, Peter Kampmann, Torben Bridstrup Pedersen

**Affiliations:** 1 Department of Clinical Physiology, Nuclear Medicine & PET, Rigshospitalet, Copenhagen University Hospital, Blegdamsvej 9, DK-2100 Copenhagen, Denmark; 2 Department of Pathology, Rigshospitalet, Copenhagen University Hospital, Blegdamsvej 9, DK-2100 Copenhagen, Denmark; lene.sjoe@regionh.dk; 3 Department of Haematology, Rigshospitalet, Copenhagen University Hospital, Blegdamsvej 9, DK-2100 Copenhagen, Denmark; Peter.Kampmann@regionh.dk; 4 Department of Surgery, Herlev Hospital, Borgmester Ib Juuls Vej 1, DK-2730 Herlev, Denmark; torben.pedersen.01@regionh.dk

**Keywords:** histiocytic sarcoma, hematologic neoplasm, hybrid imaging, 2-[^18^F]FDG PET/CT

## Abstract

Histiocytic sarcoma (HS) is a rare hematopoietic neoplasm derived from non-Langerhans histiocytic cells of the monocyte/macrophage system. With an incidence of 0.17/million individuals and a slight male preference, HS presents with a wide age distribution. Most commonly, it occurs as a primary malignancy. In approximately 25% of the cases a presumed transdifferentiation of a preexisting hematolymphoid disorder can be demonstrated. The clinical presentation varies from a localized solitary mass to severe disseminated disease often with extranodal involvement including skin, soft tissue, the gastrointestinal tract and the hematopoietic system. Systemic symptoms in terms of weight loss, fever and night sweats often occur. The diagnostic work-up of HS is extremely challenging due to the rarity of the disease as well as a wide differential diagnosis in terms of a histologic overlap with diverse mimics. No standardized treatment for HS exists and especially in a disseminated disease the clinical course is overly aggressive with a dismal outcome. The median overall survival from the time of diagnosis is approximately six months. We report a 43-year-old previously healthy Caucasian male admitted to our hospitals with abdominal pain and a feeling of fatigue. We demonstrate both the challenges of a correct diagnosis and an effective treatment as well as the aggressive nature of histiocytic sarcoma.

## Introduction

**Figure 1:**
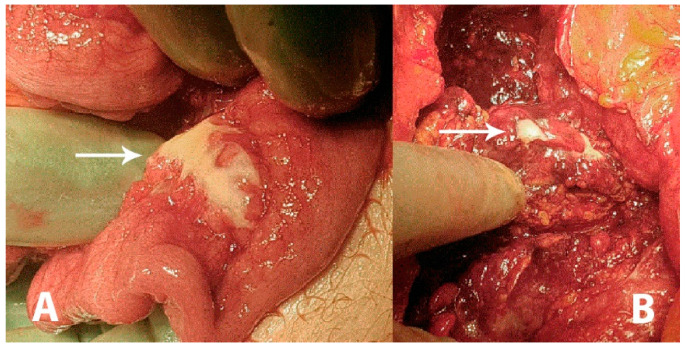
A 43-year-old previously healthy male was admitted to the surgical unit by his general practitioner with suspicions of acute diverticulitis. The patient complained of constant pain in the epigastric region and under the right and left rib curvature for the previous week upon admission. The pain was intensified when bending forwards and accompanied by an unspecific feeling of fatigue. The patient reported similar symptoms three weeks prior to admission, which remitted spontaneously. Any systemic symptoms of fever, unintended weight loss or night sweats were denied. At admission, the patient had a slight tachycardia; all other vital parameters were normal. Initial laboratory data came out with an elevated C-reactive protein (CRP; 176 mg/L) and a slightly elevated total leukocyte count (9.8 × 10^9^/L). Hepatic and renal biochemical markers were within normal ranges. On physical examination the abdomen was described as tender in the upper quadrants and around the umbilicus but without peritoneal reaction. On suspicion of an intraabdominal abscess, a computed tomography (CT) scan was performed showing free liquid surrounding the liver and the spleen as well as in the small pelvis. There was no free air. However, the upper abdomen was seen with a diffuse reaction in the fatty tissue around the pylorus and ventricle. Radiologically, a perforated ulcer was suspected. The patient underwent a diagnostic laparoscopy, revealing peritonitis in all quadrants and three liters of free fluid. There were no signs of feces, pus or bile. The greater omentum was adherent in a conglomerate consisting of the duodenum, ventricle, pancreas and transverse colon. It was not possible to get a sufficient overview of the structures laparoscopically and the procedure was converted to an explorative laparotomy revealing reactive, stearin-like changes spread in the peritoneal cavity ((**A**,**B**), white arrows). The conglomerate was dissected and the posterior part of the ventricle was inspected, revealing no ulcers. The duodenum was mobilized by the Kocher maneuver, also revealing no ulcers or perforation. Perioperatively, an esophago-gastro-duodenoscopy was performed, revealing no ulcerations or pathology. A piece of the omentum was removed for histopathological examination and an intraabdominal drain was placed before ending the procedure. The findings were suspected to be caused by pancreatitis. The postoperative course was uneventful, the drain was removed after seven days and the patient was discharged after eight days with a plan of outpatient follow-up. On postoperative day 17 he was readmitted for drainage of 3.5 L ascites, which had recollected. The pancreas was without signs of pancreatitis on a triple-phase CT. Diagnostic ultrasound described no gallstones.

**Figure 2:**
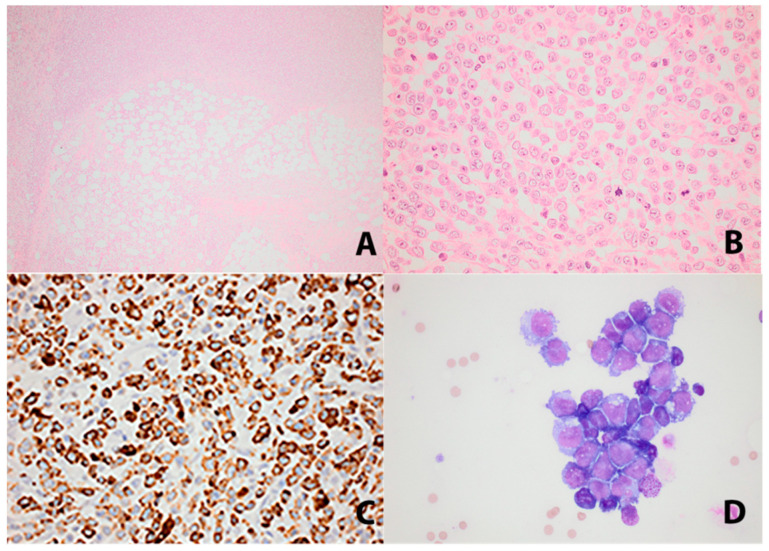
Histopathology of omental infiltrate and ascitic fluid: (**A**) Omental fatty tissue with diffuse infiltration of tumor cells (HE×4). (**B**) The tumor cells were large with vesicular nuclei. Frequent mitoses were seen (HE×40). (**C**) CD68PGM1 showing characteristic granular cytoplasmic staining (×40). (**D**) Cytomorphology of the tumor cells in ascitic fluid (×40). A histopathological examination of the resected tissue from the omentum demonstrated a dense and diffuse infiltration of the fatty tissue by large mononuclear cells with large vesicular nuclei with eosinophilic nucleoli. The cytoplasm was eosinophilic with some vacuolization. The cells were of a hematological origin and expressed CD45. They had a fully developed histiocytic differentiation profile with an immunohistochemical expression of CD68, CD163, CD4, CD56 and lysozyme and showed a high proliferative rate that in areas reached 50% (Ki-67). The precursor cell, dendritic cell, melanocytic, neuroendocrine cell, B-cell, T-cell and Langerhans cell markers were negative. No BRAF-mutation could be demonstrated. The PD-L1 was negative. Next generation sequencing (NGS) could not demonstrate any fusions. The NGS panel used was ‘Archers FusionPlex Lymphoma’, investigating the mutation, expression and fusion of 125 genes. Ascitic fluid cytology revealed malignant cells with the same phenotype as the tumor tissue in the omentum. The findings were most compatible with histiocytic sarcoma (HS). A differential diagnosis could be monoblastic leukemia, which was excluded on the examination of bone marrow biopsies. It took 24 days from initial hospitalization until diagnosis, which was confirmed by an expert second opinion. The following day the patient was transferred to a specialized hematological department at a tertiary hospital.

**Figure 3:**
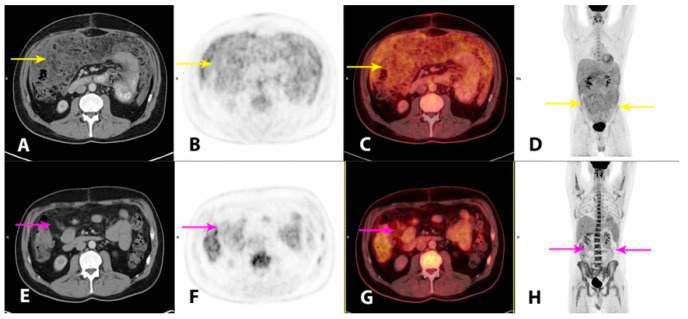
Hybrid imaging with 2-deoxy-2-[^18^F]fluoro-D-glucose positron emission tomography/CT (2-[^18^F]FDG PET/CT) was performed for the staging of the disease and as baseline for monitoring of the treatment response. Imaging performed 1 h post-injection of 4.0 MBq/kg 2-[^18^F]FDG showed a moderately metabolically active, irregular thickening of the peritoneal folds and extensive carcinomatosis-like infiltration in the omentum, mesentery and the pelvic cavity (**A**–**D**, yellow arrows). There was minor ascitic fluid in the abdominal and pelvic cavities. There were also metabolically active, enlarged lymph nodes in the mediastinum and upper retroperitoneum. The findings were considered highly suspicious for malignancy. At this point, plasma lactate dehydrogenase (LDH) levels, a future tumor marker in this patient, were elevated (366 U/L; reference range 105–205 U/L). An initial steroid treatment with prednisolone 100 mg/daily had a good effect on the tendency of the recollection of ascites. However, as no standardized treatment for this patient category without comorbidity exists, a hematological specialized tumor board, after consent from the patient, decided to administer a chemotherapy regimen of ifosfamide, carboplatin and etoposide (ICE), which was initiated one month after initial hospitalization. The patient tolerated the treatment well. At clinical follow-up after two cycles of ICE, the patient’s wellbeing was improved, the plasma LDH was normalized and imaging with 2-[^18^F]FDG PET/CT demonstrated a substantial partial response (PR), both metabolically and morphologically, of the previously described findings in the peritoneum. There were still metabolically active, carcinomatosis-like findings in the omentum, mesentery and on the liver surface (**E**–**H**, magenta arrows). There were no pathological lymph nodes. After completion of four cycles of ICE, the metabolic complete response (CR) and a further morphological PR were reported on follow-up 2-[^18^F]FDG PET/CT (not shown). The plasma LDH was still within a normal range. The patient tolerated the fifth and final cycle of ICE well.

**Figure 4:**
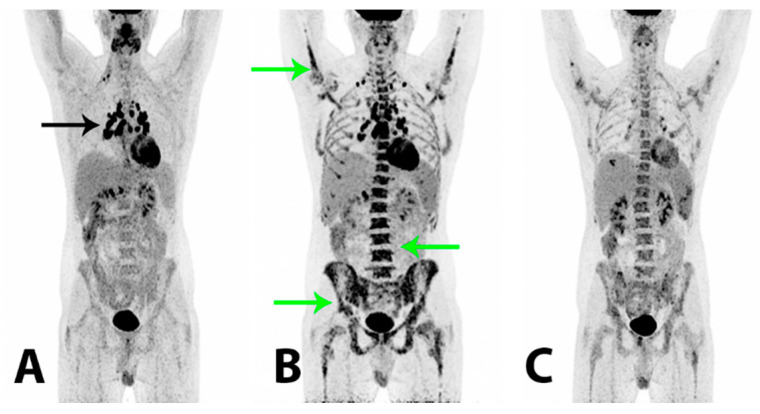
To reduce the risk of HS relapse, an experimental therapeutic approach with high-dose chemotherapy administering a BEAM (carmustine + etoposide + cytarabine + melphalan) regimen and a subsequent autologous stem cell transplant (SCT) was initiated. The patient tolerated the treatment well and at follow-up +60 days after BEAM/SCT the regeneration of bone marrow was considered complete and the tumor marker plasma LDH was still within the reference range (157 U/L). 2-[^18^F]FDG PET/CT (**A**) demonstrated a metabolic CR and a further morphological PR. There were now relatively symmetrically localized, metabolically active lymph nodes at the right root of the neck, in both sides of the mediastinum and the lung hilum (black arrow), compatible with possible granulomatous inflammation. Two months later the patient reported a gradual worsening of bone pain in the lumbar and pelvic region. The plasma LDH was now elevated (450 U/L) and thrombocyte and hemoglobin levels were low. A relapse of HS was suspected, which was confirmed by a bone marrow biopsy that showed a massive infiltration of almost 100% of malignant histiocytic cells with blastoid morphology and an immunohistochemical profile similar to previous specimens. Findings on 2-[^18^F]FDG PET/CT (**B**) were compatible with a relapse, demonstrating a heterogeneous, pathologically increased FDG-uptake in the bone marrow in the axial and peripheral skeleton (green arrows). There were metabolically active lymph nodes above and now also below the diaphragm. A malignant bone marrow infiltration with a myeloid expansion was considered the cause of the patient’s bone pain. The plasma LDH rose rapidly to 5100 U/L and salvage therapy with an acute myelogenous leukemia regime (CLAG-M; cladribine + cytarabine + mitoxantrone) was initiated. The treatment had a good effect on the patient’s bone pain and wellbeing. The plasma LDH was normalized within days and histopathological examinations showed no tumor cells in the blood and no malignant cells in the bone marrow biopsy. 2-[^18^F]FDG PET/CT (**C**) demonstrated a metabolic PR of previously malignant findings in the bone marrow. There were now splenic lesions compatible with abscesses. A second course of CLAG-M was administered and, as the patient had achieved a second CR confirmed by a bone marrow biopsy, a non-myeloablative allogeneic SCT was considered. However, within a short period of time the patient again reported bone pain and he developed cutaneous papules on the trunk end lower extremity from which a biopsy showed HS. The plasma LDH also increased rapidly and 2-[^18^F]FDG PET/CT (not shown) again demonstrated progression with a pathologically increased FDG-uptake in the bone marrow and new metabolically active cutaneous lesions on the abdomen. The findings were compatible with a systemic relapse and an allogeneic SCT was now not a feasible treatment option. Palliative treatment with a modified FLAG-Ida regime (fludarabine + cytarabine + idarubicin + filgrastim) initially had a good effect on the bone pain; the cutaneous lesions regressed and the plasma LDH decreased. However, the effect was short-lived and the patient experienced intermittent fever, bone pain, fatigue and blood cell counts that demonstrated cytopenia. The plasma LDH again increased. The patient’s clinical condition worsened significantly and after short periods of treatment with high-dose cytarabine and hydroxycarbamide, further treatment attempts with intensive regimen chemotherapy were considered futile. No appropriate protocolled treatment was found suitable for the patient and the treatment aim of long term survival was abated. The patient passed away shortly after, 15 months after his initial hospitalization.

Histiocytic sarcoma is a rare hematopoietic neoplasm derived from non-Langerhans histiocytic cells of the monocyte/macrophage system [1,2]. With an incidence of 0.17/million individuals and a slight male preference, HS presents with a wide age distribution [1,3,4,5]. Most commonly, it occurs as a primary malignancy; however, in approximately 25% of the cases a presumed transdifferentiation of a preexisting hematolymphoid disorder can be demonstrated [6]. The clinical presentation varies from a localized solitary mass to a severely disseminated disease often with extranodal involvement including skin, soft tissue, the gastrointestinal tract and the hematopoietic system [7]. Systemic symptoms in terms of weight loss, fever and night sweats often occur [8]. The diagnostic work-up of HS is extremely challenging due to the rarity of the disease as well as a wide differential diagnosis in terms of a histologic overlap with diverse mimics [7]. No standardized treatment for HS exists and especially in a disseminated disease the clinical course is overly aggressive with a dismal outcome. The median overall survival from the time of diagnosis is approximately six months [4]. We report a 43-year-old previously healthy Caucasian male admitted to our hospitals with abdominal pain and a feeling of fatigue. We demonstrate both the challenges of a correct diagnosis and an effective treatment as well as the aggressive nature of histiocytic sarcoma (Figure 1, Figure 2, Figure 3 and Figure 4).

To summarize, this case clearly demonstrates the challenging course of patients with histiocytic sarcoma. The rareness and severe complexity of HS requires a multidisciplinary and specialized approach throughout the diagnostic workout, treatment and follow-up. A rapid establishment of a correct diagnosis, staging of the disease and a personalized, aggressive treatment strategy is essential to improve outcomes in these patients. Even so, the prognosis remains extremely dismal, especially in the case of a disseminated disease at the time of diagnosis.

## Data Availability

Not applicable.
